# Clinical Assessment of the Relationship of Dental Implant Materials (Titanium and Zirconia) and Peri-Implantitis: A Systematic Review

**DOI:** 10.1007/s12663-024-02409-9

**Published:** 2024-12-10

**Authors:** Deepti Shrivastava, Syed Altafuddin Quadri, Abdulkhaliq Ali F. Alshadidi, Ravinder Saini, Meghna Dewan, Gustavo Vicentis Oliveira Fernandes, Kumar Chandan Srivastava

**Affiliations:** 1 Department of Preventive Dentistry, College of Dentistry, Jouf University, Sakaka, Saudi Arabia; 2 Department of Dental Technology, COAMS, King Khalid University, Abha, Saudi Arabia; 3 Sudha Rastogi College of Dental Sciences and Research, Faridabad, 121002 India; 4 A. T. Still University – Missouri School of Dentistry & Oral Health, St. Louis, MO 63104 USA; 5 Department of Oral & Maxillofacial Surgery & Diagnostic Sciences, College of Dentistry, Jouf University, Sakaka, Saudi Arabia; 6 Department of Oral Medicine and Radiology, Saveetha Dental College, Saveetha Institute of Medical and Technical Sciences, Saveetha University, Chennai, Tamil Nadu India; 7 All India Institute of Medical Sciences, New Delhi, India

**Keywords:** Peri-implantitis, Risk factors, Titanium, Zirconia, Dental implant, Systematic review

## Abstract

**Objective:**

This study analyzed clinical parameters to assess whether dental implant material is a risk factor for peri-implantitis.

**Methods:**

A literature search was performed on PubMed Central, Cochrane, PubMed/MEDLINE, Embase, and Scopus. The PICO strategy involved healthy patient, partially or fully edentulous, receiving at least one dental implant; zirconia or titanium dental implants; comparison involving assessment of whether there were differences in the risk of peri-implantitis among different materials used for dental implants; clinical parameters. Quality assessment was performed using the modified Jadad scale.

**Results:**

Nineteen articles met the inclusion criteria. BoP did not have statistically significant differences comparing zirconia and titanium implants or natural teeth. MBL had diversified results; sometimes, it was higher in zirconia implants than titanium; otherwise, there was no significant difference. Comparing implants with natural teeth, MBL was lower in titanium implants over prolonged observation periods, and greater severity was found in the zirconia group. Notably, natural teeth had minimal bone loss. Zirconia implants demonstrated reduced plaque accumulation and minimal microbial contamination compared to titanium implants and control teeth. The quality assessment was considered poor to low in 9 studies and good to excellent in 10. The development of peri-implantitis was influenced by several patient-specific and clinical factors, underscoring the need to adopt a comprehensive and personalized approach to implant dentistry and peri-implantitis prevention.

**Conclusion:**

It was not possible to draft any solid conclusion for the relationship between implant material and peri-implantitis.

## Introduction

Dental implants have significantly transformed Dentistry by offering dependable and long-lasting alternatives for restoring missing teeth [1]. The selection of the implant material is crucial in achieving dental implant success [2]. Since its introduction over forty years ago, commercially pure titanium has been considered the gold standard for intraosseous dental implants [3]. Whereas titanium grade 4 is used in most titanium dental implants, titanium grade 5 is used in titanium alloys, such as Ti-6Al-4 V, which have improved fatigue resistance and strength [4].

Despite titanium's advantageous mechanical properties and biocompatibility, certain disadvantages exist, including the risk of hypersensitivity reactions and peri-implant soft tissue discoloration [5]. Moreover, titanium debris may be detached from the implant surface upon placement. It can trigger DNA damage response signaling in oral epithelial cells, which can disrupt epithelial homeostasis and potentially compromise the oral epithelial barrier [6]. Also, titanium exposure to fluoride or metal alloys in saliva has been shown in earlier research to cause a corrosion event [7]. It has also been suggested that bacterial biofilms may cause titanium implant surface oxidation in an acidic environment, which would trigger inflammatory responses [3]. Zirconia implants have recently emerged as viable alternatives, offering numerous potential benefits and mitigating specific titanium-related challenges [8, 9]. They exhibit enhanced toughness and fracture resistance owing to allotropy, which leads to phase transformation toughening mechanisms [10]. In addition to their exceptional esthetic characteristics, zirconia implants exhibit biocompatibility comparable to titanium implants and reduced plaque affinity [11].

The selection of implant material is determined by various factors, such as biocompatibility, mechanical characteristics, esthetics, and the host's reaction to the material [2, 12]. Furthermore, an important aspect in determining the efficacy of dental implants is the presence of bacterial colonization surrounding them [13]. The type and quantity of bacteria at the abutment–implant contact are essential factors in determining how well the peri-implant tissues function [13, 14]. A modified microbial composition could potentially increase the susceptibility of implants to peri-implantitis [15]. The clinical outcomes of dental implants are significantly influenced by the interplay between inflammatory responses, immunological reactions, and the ability of implant materials to regulate these processes [15].

Peri-implantitis is a peri-implant disease characterized by inflammation and loss of bone tissue surrounding dental implants. It is a crucial and crescent concern in implant dentistry [16, 17]. Gaining insight into the risk factors for peri-implantitis and their association with dental implant materials (titanium and zirconia) is paramount to clinicians [16, 18]. However, identifying risk factors can aid in early detection and timely intervention. Prioritizing peri-implantitis is paramount to achieve effective treatment outcomes and enhance the overall prognosis. Then, dental professionals can closely monitor patients more susceptible to this condition. Previous systematic reviews focused only on titanium and zirconia implants' success/survival rate [3, 8, 19] and their clinical parameters [20]. Then, this review aimed to determine whether dental implant composition increases the risk of peri-implantitis. Thus, the study's objectives were to assess the impact of dental implant material on peri-implantitis, correlating the material used with the likelihood of developing this disease. The null hypothesis was no one material presented a cause specificity correlation for peri-implantitis development.

## Materials and Methods

The preferred reporting items for systematic reviews and meta-analyses (PRISMA) standards were the foundation for constructing the systematic review's framework. The international platform of registered systematic review and meta-analysis protocols (INPLASY) was the registered protocol for this systematic review (202,350,042). This study followed the Preferred Reporting Items for Systematic Review and Meta-Analysis (PRISMA) criteria [21]. The PICO framework [22] was defined as follows to assess whether dental implant material is a risk factor for peri-implantitis: population (*P*): systematically healthy, partially or fully edentulous, receiving at least one dental implant; intervention (*I*): zirconia or titanium dental implants; comparison (*C*): comparison involving assessment of whether there were differences in the risk of peri-implantitis among different materials used for dental implants (zirconia vs. titanium implants); outcome (*O*): (i) primary outcomes: Bleeding on Probing (BoP), marginal bone loss (MBL), plaque index (PI), and the secondary outcome was the implant survival rate (SR).

### Search Strategy

A detailed search on PubMed Central, Cochrane, PubMed/MedLine, Embase, and Scopus was performed up to October 4, 2023. A search was performed to retrieve scholarly articles that explored whether dental implant material is a risk factor for peri-implantitis. The following combinations of phrases were applied, with modulation according to the database: ("Dental implant materials" OR "Dental implants" OR "Tooth implants" OR "Dental prostheses" OR "Zirconia" OR "Titanium") AND ("Peri-implantitis" OR "Peri-implant infection" OR "Implant failure" OR "Dental implant failure" OR "Osseointegration failure") AND ("Prevalence" OR "Incidence" OR "Epidemiology" OR "Occurrence" OR "risk factors"). The key terms were comprehensively combined in multiple databases.

### Inclusion and Exclusion Criteria

In order for a study to be deemed suitable for this evaluation, it had to fulfill the subsequent requirements: Clinical study published in peer-reviewed journals; studies available in the English language; studies focused on the impact of dental implant material, and the risk factors for peri-implantitis. Studies were excluded for the following reasons: reviews, meta-analyses, abstracts, comments, or editorial articles; they had no methods or results evaluating peri-implantitis and possible risk factors; in vitro studies; and studies conducted on animal models.

### Study Selection

After completing the initial search strategy, the articles were selected using a methodical and sequential approach. The Zotero reference manager (https://www.zotero.org/) was used to eliminate duplicate studies, and the remnant studies were subsequently incorporated into the screening phases for review. The screening was performed by two independent authors (DS and SAQ), and in case of divergence, a third author (GVOF) was consulted. A comprehensive evaluation was conducted on all potential articles. Articles not fulfilling the qualifying criteria were excluded during the first screening step, which entailed evaluating titles and abstracts. The full texts of the remaining articles were evaluated using preset criteria for inclusion and exclusion. Accuracy was assessed for articles that fulfilled all inclusion criteria.

### Data Extraction and Data Synthesis Methods

Two independent researchers (DS and AAFA) performed data extraction. In case of disagreement, another author was invited to untie. Then, the data were compiled into tables (Microsoft® Excel, v. 16.50, Microsoft Office, Redmond, WA, USA) based on the authors, year of publication, the research design, the sample size, details about the dental implant, report about peri-implantitis, information about the patients, follow-up period, implant survival rate, and clinical information (MBL, PI, and BoP).

### Quality Assessment

Each study was assessed using the modified Jadad scale [23]. This eight-item scale evaluated randomized, blinding, withdrawals and dropouts, inclusion and exclusion criteria, adverse effects, and statistical analysis. The score for each article could range from 0 (lowest quality) to 8 (highest quality). Scores of 4–8 denote good to excellent quality and 0–3 poor to low quality. Critical appraisal was conducted by one author (DS) and was verified by another (GVOF).

## Results

### Selection Process

**Fig. 1:**
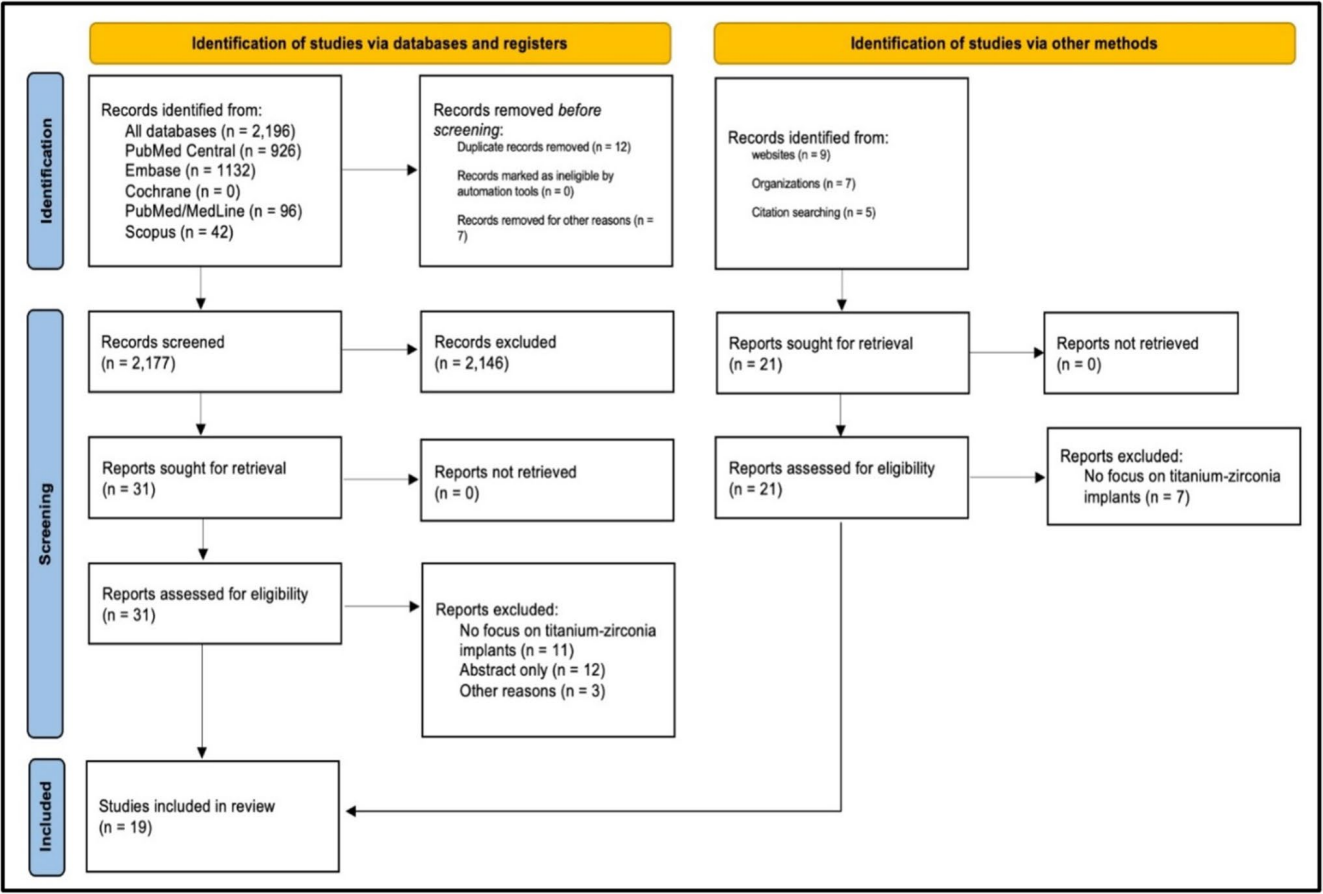
PRISMA flowchart for the screening and selection of articles

The search yielded 2196 potential articles, of which 12 duplicates were removed along with seven other articles removed for other reasons. The exclusion criteria, title, and abstract screening excluded 2146 articles. A total of 52 publications were retrieved and subsequently evaluated for eligibility. Thirty-three publications were excluded from the analysis to adhere to the predetermined criteria for inclusion. Nineteen studies [13, 24–41] were deemed eligible for review after screening, as depicted in Fig. 1.

### Main Studies’ Characteristics

Table 1 provides a summary of the outcome measures and significant findings. Eight hundred eighty-seven participants were included (most were female adults), with a mean age of over 50 years. The minimum average follow-up duration was six months, and the maximum was 20 years. The implant materials used were single- or two-piece zirconia, titanium, or alloys.

**Table 1:** Summary of outcome measures and significant findings

Authors	Study design	*N* participants and *N* implant	Participant characteristics	Follow-up	Implant material used	Methods used	Implant success/survival rate	Bleeding on probing (BoP)	Marginal bone loss (MBL)	Plaque index (PI)	Insights	Results
Schwarz et al. (2015) [31]	Prospective case series-non-surgical treatment of peri-implant mucositis and peri-implantitis	34 patients 45 implants	Not provided	6 months	Zirconia	Mechanical debridement + local antiseptic therapy and Er: YAG laser therapy	52.9% for peri-implant mucositis treatment 29.4% for peri-implantitis treatment	Ranged from 14.3–47.5	Not Reported	No significant differences in plaque index were reported	–	Non-surgical treatment showed clinical improvements Disease resolution was achieved in 52.9% of peri-implant mucositis patients and 29.4% of peri-implantitis patients
Kniha et al. (2018) [36]	Retrospective study	86 individuals 123 zirconia implants	Not provided	1 year	Zirconia implants (Straumann PURE Ceramic Implant)	Radiographic investigation at different time points	94.5%	No significant difference between groups	No occurrence of substantial bone loss around the implant	–	No significant peri-implant bone loss was observed in both periodontally healthy and compromised patients	The survival rate was 100% No significant peri-implant bone loss was observed in the first year
Holländer et al. (2016) [32]	Clinical study	38 adults 106 zirconia implants	18 M/20F Mean age = 56.24 Age range = 33–74	1 year	Zirconia	Assessment of plaque index, bleeding, pocket depth	Survival rate was 100% There was no discernible difference between dental implants and natural teeth, according to the data	No statistical significance observed	Not mentioned in the study	The zirconia implant group had much lower plaque index than natural teeth	The study found that zirconia dental implants had similar clinical results to natural teeth and were well accepted by patients	Zirconia dental implants showed similar clinical outcomes to natural teeth The implant group had greater colonization of bacteria associated with periodontitis and peri-implantitis (not statistically significant)
Lorenz et al. (2019) [13]	Prospective longitudinal clinical study	28 adults 83 zirconia implants	13 M/15F Mean age = 63.5 Age range = 39–80	7.8 years	Zirconia	Analysis of clinical parameters (SBI, PPD, REC) Microbial analysis using Paro Check 20	100% survival rate of zirconia implants	No statistical significance was observed between implants and teeth	Mean MBL of 1.2 mm Moderate bone resorption without indication for peri-implantitis	Zirconia implants showed significantly less plaque accumulation The plaque index was analyzed and found to be lower in the study group	The study found favorable long-term clinical results for zirconia implants, with high patient satisfaction and some microbial contamination	The survival rate of zirconia implants was 100% One implant presented a profound peri-implantitis resistant to therapies The microbial analysis revealed no statistically significant colonization of periodontitis/peri-implantitis bacteria in the implant group
Rodriguez et al. (2018) [37]	Retrospective case series	12 participants 24 implants	5 M/7F Mean age = 55 Age range = 27–86	25 months	Zirconia dental implants were used in the study	Gingival index and Plaque index	The success rate was 92%	Not reported in the study	The present clinical evaluation showed a mean peri-implant bone loss of 0.3 mm 33.3% of the implants had radiographic detectable peri-implant bone loss	Ranged from 0 to 1 Low plaque accumulation was observed in zirconia implants	The clinical evaluation of zirconia dental implants showed a success rate of 92% with minimal peri-implant bone loss	Overall success rate of zirconia implants: 92% Zirconia implants showed low plaque accumulation
Borgonovo et al. (2015) [30]	Retrospective study	13 patients 20 implants	13 M/1F Mean age = 60	4 years	Zirconia implants were used in the study	Clinical and radiographic evaluation performed at regular intervals	The success rate = 100%	Not provided	Bone loss of 2.1 mm MBL reduction due to one-piece morphology of zirconia implants	Median and mode were both equal to 1 Zirconia dental implants have less plaque	The study evaluated the survival and success rates, marginal bone loss, and periodontal indexes of zirconia implants in esthetic areas over four years	100% success rate Bone loss of 2.1 mm Zirconia dental implants have low plaque adhesion
Kohal et al. (2018) [34]	Prospective controlled study	65 patients (one-stage implant surgery)	25 M/40F	3 years	Zirconia implants used in the study	1-stage implant surgery with immediate temporization Standardized radiographs taken at different time points	Cumulative survival rate of 90.8% 6 posterior site implants were lost	Increased	Mean MBL: 1.45 mm 35% of implants lost 2 mm of bone	Decreased	Clinical and radiological effects of single-tooth replacement with one-piece zirconia	Cumulative survival rate of 90.8% after three years 35% of implants lost at least 2 mm of bone 22% lost 3 mm of bone
Venza et al. (2009) [25]	RCT	90	12 M/44F Age range = 30–60	3 years	Titanium implants	Examination of patients with submerged and non-submerged implants Evaluation of PI, gingival index, PPD, CAL, and MBL	The study compared submerged and non-submerged implants Submerged implants had higher risks for periodontal complications	Not provided	MBL was significantly higher in the submerged group compared to the non-submerged group There was a slight increase in bone loss at 24 months	PI was evaluated in the study It was significantly higher in the submerged group	Submerged implants had higher levels of inflammatory mediators in the peri-implant fluid than non-submerged implants	Submerged implants have a higher risk of periodontal complications—Inflammatory mediator varied in peri-implant fluid, which can indicate elevated risk
Delucchi et al. (2021) [40]	Prospective clinical study	18 patients 42 implants	–	9.3 years	Titanium implants	Dual acid-etched (DAE) surface treatment Machined surface treatment	No implant failures were reported Success rate not explicitly mentioned in the text	There were no statistically significant differences for BoP No significant effect of titanium surface treatments on BoP	Moderate crestal bone remodeling occurred during the first year after implant insertion	PI was recorded during the study It was not significantly different	The study found that titanium implants with a DAE surface reduced peri-implant bone loss in the initial healing phase compared to machined surfaces	No statistically significant differences in peri-implantitis were found DAE surfaces reduce peri-implant bone loss Minimally rough surfaces favor peri-implant bone maintenance
Chappuis et al. (2013) [26]	Prospective clinical study	67 patients 95 implants	31 M/36F Mean age = 66.3 Age range = 39–95	20 years	Titanium plasma-sprayed (TPS) surface	67 patients with fixed dental prostheses were examined Implants were classified as successful, surviving, or failed	Survival rate: 89.5% 20-year implant success rate: 75.8%	Not provided	Implant survival rate was 89.5%. In 92% of implants, radiographs showed crestal bone loss under 1 mm Only 8% showed peri-implant bone loss > 1 mm No one had severe bone loss greater than 1.8 mm During observation, 19 implants had biological complications with suppuration. Among 19 implants, 13.7% were successfully maintained after treatment for 20 years Technical problems were observed in 32%	Not provided	92% of implants had crestal bone loss below 1 mm 8% of implants had peri-implant bone loss > 1 mm	Implant survival rate of 89.5% after 20 years 75.8% implant success rate after 20 years 8% of implants exhibited peri-implant bone loss > 1 mm None of the implants exhibited severe bone loss > 1.8 mm
Karoussis et al. (2004) [24]	Prospective cohort study	89 patients 179 implants (112 hollow screws, 49 hollow cylinders, 18 angulated hollow cylinders)	31 M/36F Mean age = 66.3 Age range = 39–95	10 years	Titanium implants	Clinical and radiographical parameters	Success rates ranged from 61% to 90.2% Hollow screw implants had a significantly higher success rate than hollow cylinder implants	It was one of the clinical parameters assessed	Not provided	PI used to evaluate plaque accumulation on implants	A significantly higher success rate and a significantly lower incidence of peri-implantitis were identified for hollow screw design ITI Dental Implants after ten years compared to hollow cylinder design ITI dental implants	Hollow screw design implants had a higher survival rate (95.4%) Hollow cylinder design implants had a higher incidence of peri-implantitis (29%)
Rasul et al. (2021) [41]	Clinical study	60 patients	Not provided	Not provided	Titanium implants	Inductively coupled plasma mass spectrometry Probing depth, gingival index, plaque index, plaque mass	Not provided	0.64 ± 0.3 in Group I 1.64 ± 0.8 in Group II	Not provided	The mean plaque index in Group I (peri-implantitis) was 0.82 ± 0.2 The mean plaque index in Group II (healthy implants) was 1.5 ± 0.6	The study found a significantly higher titanium level in submucosal plaque around peri-implantitis than in healthy implants	Probing depth and gingival index were higher in Group II (peri-implantitis) Titanium level was significantly higher in Group II (peri-implantitis)
Safioti et al. (2017) [35]	Cross sectional Study	30 participants	Not provided	Not provided	Titanium implants	Submucosal plaque collected from implants with peri-implantitis and healthy implants Levels of titanium quantified using inductively coupled plasma mass spectrometry	Not provided	Not provided	Not provided	–	The study found an association between dissolved titanium levels and peri-implantitis in dental implants	Dissolved titanium levels were higher in implants with peri-implantitis Corrosion of titanium surfaces may aggravate inflammatory response
Horikawa et al. (2017) [33]	Retrospective cohort study	92 patients 223 implants	40–60 years old	At least 25 years after the prosthesis’ installation	Titanium implants (plasma-sprayed surfaces)	Retrospective analysis of patient cases Kaplan–Meier survival curves used for calculations	Survival rate of 89.8% after 25 years of functioning The survival rate of maxillary positioned implants was significantly lower than that of mandibular positioned implants The main reason for implant failure was peri-implantitis, with 1416 failed implants	Not provided	Not provided	Not provided	The study found that the cumulative incidence of peri-implantitis in titanium implants of 15.3% at 10 years, 21.0% at 15 years, and 27.9% at 25 years	Cumulative survival rates of implants at 10, 15, and 25 years were 97.4%, 95.4%, and 89.8%, respectively, Cumulative incidences of peri-implantitis at 10, 15, and 25 years were 15.3%, 21.0%, and 27.9%, respectively,
Osman et al. (2013) [27]	RCT	24 participants 168 implants	15 M/4F Mean age = 62 Age range = 46–80	1 year	One-piece titanium implants One-piece zirconia implants	Assessment of clinical parameters	No significant difference in survival rate between titanium and zirconia implants Zirconia implants had higher fracture rates and bone loss		Around titanium implants (0.18 mm) Zirconia implants had a higher MBL (0.42 mm)	–	–	Titanium implants had higher survival rates and lower bone loss Peri-implant MBL was evaluated Peri-implant soft tissues showed a significant increase in PPD, modified plaque index (mPI), and modified bleeding index (mBI)
Siddiqi et al. (2013) [28]	RCT	24 participants (12 Ti, 12 Zr) 150 implants (Ti and Zr)	15 M/4F Mean age = 62 Age range = 50–79	1 year	Titanium implants Zirconia implants	Use of titanium and zirconia implants with different surface characteristics	Success rates for both groups were low Only 11 implants (52.4%) out of 21 palatal implants survived over the follow-up	Peri-implant health was equivalent between groups Comparable soft tissue response around implants	Zirconia implants showed greater bone loss than titanium implants No statistically significant differences in peri-implant bone levels	Participants showed improvements in plaque index	The study found that zirconia and titanium implants had low success rates, with zirconia implants showing greater bone loss	Failure rates were higher with one-piece zirconia implants compared to titanium implants No statistically significant differences in peri-implant health No sign of peri-implant pathology was observed
Bienz et al. (2021) [39]	RCT	42 patients Each patient received one zirconia and one titanium implant	–	3 months	Zirconia implants Titanium implants	Clinical parameters were evaluated before and after the experimental phase	Zirconia implants had lower plaque and bleeding scores under experimental mucositis conditions	Increased significantly in the titanium group Remained stable in the zirconia group	Not provided	Plaque control record increased significantly for both implants under experimental mucositis conditions Zirconia implants had lower plaque scores compared to titanium implants	The study observed reduced levels of plaque and bleeding in zirconia implants when subjected to experimental mucositis conditions Both zirconia and titanium implants had similar outcomes under healthy conditions	Zirconia implants had lower plaque and bleeding scores under experimental mucositis conditions No significant histological differences were observed between the two implant types
Payer et al. (2014) [29]	RCT	22 patients 31 implants (16 zirconia and 15 titanium)	13 M/9F Mean age = 46 Age range = 24–77	24 months	Zirconia implants Titanium implants	Two-piece zirconia implants and titanium implants	93.3% for zirconia 100% for titanium	It was assessed during clinical examinations	MBL was observed in zirconia and titanium implants No cases of peri-implantitis were reported	Not provided	Two-piece zirconia implants were compared to titanium implants	Success rates of zirconia implants were comparable to titanium implants after 24 months No cases of peri-implantitis were reported
Koller et al. (2020) [38]	RCT	22 healthy patients 31 implants (16 zirconia and 15 titanium)	15 M/4F Mean age = 62 Age range = 50–79	80.9 months (mean). Clinical outcomes evaluated after 80 months	Two-piece zirconia implants Titanium implants	BoP, MBL, PI	There are no significant differences between zirconia and titanium implants. Limited sample size, results should be interpreted cautiously	No significant difference between groups Zirconia: 16.43% (SD: 6.16) Titanium: 12.60% (SD: 7.66)	No information about peri-implantitis was provided	Zirconia implants had 11.07% (SD: 8.11) Titanium implants had 15.20% (SD: 15.58)	The study compared all-ceramic restorations of zirconia two-piece implants with titanium implants	There are no significant differences between zirconia and titanium implants There was no significant difference in bone loss between the two types of implants

### Clinical Parameters Assessed

#### Bleeding on Probing (BoP)

For this parameter, it was found that there were no statistically significant differences in the presence of bleeding on probing (BoP) when comparing zirconia and titanium implants or compared them to natural teeth [13, 31, 36, 38, 40]. However, it is worth noting that a study conducted by Kohal et al. [34] reported increased probing depth and bleeding index over three years in a case involving a single-tooth replacement with zirconia implants. Although this finding suggests a potential adverse effect on periodontal health in this scenario, it is crucial to consider that this case was isolated and may not represent the general population.

Furthermore, various studies have observed mixed results when comparing BoP between zirconia and titanium implants. In one study, BoP increased significantly in the titanium implant group but remained stable in the zirconia implant group [39]. Conversely, another study found no significant difference in BoP between zirconia and titanium implants [38]. While the current research on this subject of BoP may be inconclusive, it is crucial to consider a multitude of elements that have the potential to impact the outcomes. These factors include the study design, sample size, implant surface characteristics, and the patient's overall health. Additionally, a comprehensive evaluation of other periodontal parameters, such as the clinical attachment level and plaque accumulation, should be considered to understand better the impact of zirconia and titanium implants on periodontal health.

#### Marginal Bone Loss (MBL)

This study's reported outcomes were diverse after comparing marginal bone loss (MBL) between zirconia and titanium implants. While some studies have shown that zirconia implants have a higher MBL than titanium implants [27, 28], other studies, such as the one conducted by Payer et al., have found no significant difference in MBL between the two materials [29]. Moreover, some studies have compared the MBL of titanium or zirconia implants with natural teeth. It was observed that titanium implants exhibited less MBL and maintained more consistent bone levels over prolonged observation periods [26]. Conversely, zirconia implants demonstrated a wide range of peri-implant bone loss severity, with some cases showing minimal MBL while others exhibited more substantial bone loss [30, 34, 37]. Notably, the control groups in these trials consisted of natural teeth, which were found to have minimal bone loss. This comparison highlights the importance of monitoring the MBL around dental implants and considering the baseline bone levels of natural teeth as a reference. These findings emphasize the need to investigate further the factors influencing MBL in zirconia and titanium implant restorations. Variables such as implant design, surface characteristics, patient-related factors, and occlusal forces may play a role in the divergent MBL outcomes observed in different studies.

#### Plaque Index (PI)

Studies examining plaque accumulation, measured by the PI, around zirconia implants have generated varying results, shedding light on the complex relationship between implant material and oral hygiene. Some research has indicated that zirconia implants exhibit lower plaque scores than titanium implants [38, 39] and control teeth [13, 32, 36, 37]. These findings suggest that zirconia implants may be advantageous for maintaining a clean oral environment. Nevertheless, it is imperative to acknowledge a lack of consistent evidence for these findings across every investigation. Some studies have reported no significant differences in plaque scores when comparing zirconia/titanium implants with control teeth [31, 40]. This discrepancy emphasizes the need for further investigation to understand the impact of zirconia implants on plaque accumulation fully. In contrast to the abovementioned observations, Rasul et al. found lower plaque accumulation in titanium implants than in control teeth [41]. This counterintuitive result highlights the complexity of the factors influencing plaque accumulation and encourages additional research to elucidate the specific mechanisms involved.

#### Quality Assessment

The quality of 9 included studies was framed between poor and low, whereas for the rest, it was considered between good and excellent (Table 2).

**Table 2:** Quality assessment using the modified Jadad score

Study	Was the study described as randomized?	Was the method of randomization appropriate?	Was the study described as blinded?	Was the method of blinding appropriate?	Was there a description of withdrawals and dropouts?	Was there a clear description of the inclusion/exclusion criteria?	Was the method used to assess adverse effects described?	Was the method of statistical analysis described?		Final score
Schwarz et al. (2015) [31]	0	0	1	1	0	1	1	1	5	
Kniha et al. (2018) [36]	0	0	0	0	0	1	1	1	3	
Holländer et al. (2016) [32]	0	0	0	0	1	1	1	1	4	
Lorenz et al. (2019) [13]	0	0	0	0	1	1	1	1	4	
Rodriguez et al. (2018) [37]	0	0	0	0	0	1	1	0	2	
Borgonovo et al. (2015) [30]	0	0	0	0	0	1	1	1	3	
Kohal et al. (2018) [34]	0	0	0	0	0	1	1	1	3	
Venza et al. (2009) [25]	0	0	0	0	0	1	1	1	3	
Delucchi et al. (2021) [40]	0	0	1	1	1	1	1	1	6	
Chappuis et al. (2013) [26]	0	0	0	0	1	0	1	1	3	
Karoussis et al. (2004) [24]	0	0	0	0	0	0	1	1	2	
Rasul et al. (2021) [41]	0	0	0	0	0	1	1	1	3	
Safioti et al. (2017) [35]	0	0	0	0	0	1	1	1	3	
Horikawa et al. (2017) [33]	0	0	0	0	1	0	1	1	3	
Osman et al. (2013) [27]	1	0	0	0	1	1	1	1	5	
Siddiqi et al. (2013) [28]	1	1	1	1	1	1	1	1	8	
Bienz et al. (2021) [39]	1	1	1	1	0	1	1	1	7	
Payer et al. (2014) [29]	1	1	0	0	0	1	1	1	5	
Koller et al. (2020) [38]	1	1	0	0	0	1	1	1	5	

## Discussion

This systematic review assessed whether dental implant material, specifically zirconia or titanium, can be a risk factor for peri-implantitis by analyzing clinical studies focused on periodontal parameters (BoP, MBL, and plaque index). Understanding this fact enables clinicians to make well-informed decisions, ensure patient safety, and improve patients' overall well-being when undergoing dental implant procedures. The findings of this review revealed that zirconia implants were associated with reduced plaque accumulation compared to titanium implants and control teeth. The clinical trial by Bienz et al. compared the soft tissue architecture surrounding zirconia and titanium implants [39]. Based on their findings, there was an observed increase in plaque control within the groups experiencing mucositis. However, an increase was significantly higher in titanium implants than in zirconia implants when subjected to experimental mucositis settings [39].

Zirconia implants have demonstrated favorable clinical outcomes, including minimal microbial contamination and reduced plaque accumulation compared to teeth [32, 36]. These findings indicate that zirconia implants have the potential to enhance plaque management and minimize plaque buildup owing to their unique material features, such as smooth surface and biocompatibility, which discourage bacterial adhesion [32, 38]. Notably, significant differences in plaque scores were not found between zirconia/titanium implants and teeth [31, 40]. The results may have been impacted by the particular attributes of the participants, including their vulnerability to plaque accumulation and their unique oral hygiene practices. Thorough dental care, adherence to oral hygiene, and implant maintenance are critical for preventing plaque-related issues around dental implants, irrespective of the implant material used.

Regarding MBL, there were significant and non-significant differences in the risk of peri-implantitis between zirconia and titanium implants. In the long run, zirconia and titanium implants showed some level of MBL, with zirconia implants showing higher amounts of bone loss. In a research project by Koller et al., over 80 months, the performance of two-piece zirconia implants was compared to titanium implants [38]. The average MBL for zirconia implants was − 1.38 mm; whereas, titanium implants had an average marginal bone loss of − 1.17 mm. This difference was not statistically significant [38]. Similar outcomes were reported by Payer et al. [29]. Conversely, other authors found that zirconia implants were associated with a greater MBL than titanium implants, and the results were statistically significant [27, 28].

Some studies have compared titanium or zirconia implants with the control group filled with natural teeth. Titanium implants exhibited less and more consistent MBL across the prolonged observation periods [26]. On the other hand, zirconia implants demonstrated a range of severity of peri-implant bone loss, whereby some cases reported less MBL while others exhibited more significant loss [30, 34, 37]. These results may be difficult to contextualize; however, titanium implants were consistently associated with less MBL than zirconia implants. These findings highlight titanium implants' enduring stability and reliability, which helps to solidify their status as the “gold standard” in Implant Dentistry. Reduced MBL in titanium implants indicates improved health of the peri-implant tissues, which is a critical factor for the success and survival of dental implants in the long-term.

Most of the included studies have reported non-significant statistical differences in the biological outcome parameter (BoP) comparing zirconia and titanium implants and also comparing zirconia/titanium implants to control teeth [13, 31, 36, 38]. However, Kohal et al. noted increased probing depth and bleeding index over three years in a single-tooth replacement utilizing zirconia implants [38]. Other studies have compared BoP between zirconia and titanium implants and found mixed results. There was a notable rise in BoP within the titanium group; whereas, the zirconia group exhibited a consistently lower level of BoP [39]. These findings underscore the significance of considering the composition of the implant material and the therapeutic procedures within the framework of BOP and the health of peri-implant tissues.

Moreover, one-piece zirconia implants demonstrated excellent short-term survival outcomes (100%). In a study conducted by Kniha et al., an examination was carried out to evaluate alterations in the peri-implant crestal bone surrounding zirconia implants in patients with both periodontal health and periodontal impairment [36]. The results revealed a 100% survival rate after a one-year follow-up period. Similar results have been previously reported [30, 32, 37]. The survival outcomes for titanium ranged from 89 to 94% over a follow-up of 10–20 years [24, 26]. These findings revealed that the survival outcomes of both titanium implants were comparable. It was also observed that detachment and repositioning of abutments may result in 0.2 mm bone loss [42].

There is a lack of evidence to confirm the role of corrosion by-products and titanium particles, a nonplaque forming factor leading to peri-implantitis. This subject was evaluated by researchers [6], who indicated that titanium debris might be detached from the implant surface upon implant placement, which can trigger DDR signaling in oral epithelial cells, contributing to the disruption of epithelial homeostasis and potentially compromising the oral epithelial barrier. Therefore, there is minimal evidence that peri-implant bone loss occurs due to a persistent foreign body reaction once osseointegration has been established [43].

Certain limitations may have influenced the findings of this study. In various studies, diverse implant thread designs, surface properties, and prosthetic superstructures have made direct comparisons difficult. It may not be entirely acceptable to compare the clinical results of implants in the midpalate region for overdenture retention with those in the edentulous ridge. To address these challenges, further research involving high-quality RCTs and CCTs is required to compare the effects of titanium and zirconia on the prevalence of peri-implantitis. To make valid comparisons, these studies must use standardized research methods, well-specified inclusion and exclusion criteria, and extended follow-up periods.

### Limitations of the Study and Potential Sources of Bias

Although these findings offer some insights, they have limitations and potential sources of bias. Some of the limitations and potential biases are as follows: (1) Tt was not possible to develop a meta-analysis because this systematic review contains research with different designs, sample sizes, patient demographics, implant types, follow-up, and techniques. Heterogeneity may cause discrepancies and complicate conclusions; (2) some studies included had small sample sizes, which reduced the statistical power and precision of the results. Small sample sizes may not adequately represent the true population characteristics and may increase the risk of obtaining spurious findings; (3) some studies have relatively short follow-up periods, which may not be sufficient to assess long-term implant success or failure. Dental implants can manifest complications or failures over extended periods, and shorter follow-up durations may not capture such events.

### Recommendations for Future Studies

The current literature on titanium and zirconia dental implants indicates several potential avenues for further research: (1) Future research endeavors should encompass long-term comparative studies with larger sample sizes to comprehensively assess the effectiveness of titanium and zirconia implants over extended durations. This will help establish the longevity and durability of both implant types and provide more comprehensive insights into their clinical outcomes; (2) researchers should be encouraged to adopt standardized methodologies, including consistent outcome measures and study protocols. This will reduce heterogeneity across studies and enhance the reliability of the results, making it easier to compare and combine the findings in future meta-analyses; (3) conduct more prospective RCTs comparing titanium and zirconia implants. RCTs are considered the “gold standard” study in research and provide robust evidence for clinical decision-making; (4) to investigate the performance of titanium and zirconia implants in specific patient groups, such as individuals with systemic diseases, compromised bone conditions, or other risk factors. This will facilitate a more comprehensive understanding of the performance characteristics of these materials in various clinical settings; (5) future studies should include patient-reported outcomes and quality-of-life assessments to gauge patient satisfaction with both implant types. This information is crucial for determining which material may provide a better overall patient experience; (6) study of how implant design and surface alterations affect titanium and zirconia implant success. Subtle implant properties may affect clinical performance; (7) data on complications and adverse events associated with titanium and zirconia implants were systematically collected to understand their safety profiles and potential risks better; (8) conduct cost-effectiveness and economic evaluations comparing titanium and zirconia implants over the long-term. This information will be valuable to healthcare providers, patients, and policymakers when making decisions regarding implant materials; (9) collaborative multicenter studies must be considered to increase the generalized ability of the findings and ensure a diverse patient population. By adhering to these study standards, dental patients and professionals can enhance dental treatment outcomes by making informed choices regarding implant materials.

## Conclusion

Within the limitation of this systematic study, it was not possible to address that the implant’s material has a possible relationship with the onset of peri-implantitis. Comparing titanium/zirconia implants to control teeth, zirconia exhibited some benefits, such as reduced plaque formation and decreased microbiological contamination. Most studies found no statistically significant differences between zirconia and titanium implants or control teeth. Furthermore, over time, zirconia and titanium implants both showed some level of marginal bone loss, with zirconia implants presenting higher bone loss. Although implant material selection may impact plaque formation and microbiological contamination, it is not the only risk factor for peri-implantitis. Many patient-specific and clinical factors may influence peri-implantitis development, highlighting the necessity of implementing an all-encompassing and individualized strategy for implant dentistry and peri-implantitis prevention.

## Data Availability

The data will be available at a reasonable request from the corresponding author.
